# Relative preservation of triceps over biceps strength in upper limb-onset ALS: the ‘split elbow’

**DOI:** 10.1136/jnnp-2018-319894

**Published:** 2019-03-07

**Authors:** Roaya Khalaf, Sarah Martin, Cathy Ellis, Rachel Burman, Jemeen Sreedharan, Christopher Shaw, P Nigel Leigh, Martin R Turner, Ammar Al-Chalabi

**Affiliations:** 1 Department of Basic and Clinical Neuroscience, Maurice Wohl Clinical Neuroscience Institute, King's College London, London, UK; 2 Department of Neurology, King's College Hospital, London, UK; 3 Department of Neuroscience, Brighton and Sussex Medical School, Sussex, UK; 4 Nuffield Department of Clinical Neurosciences, Oxford University, Oxford, UK

## Abstract

**Objective:**

Amyotrophic lateral sclerosis (ALS) is a neurodegenerative disease of the motor system. The split hand sign in ALS refers to observed preferential weakness of the lateral hand muscles, which is unexplained. One possibility is larger cortical representation of the lateral hand compared with the medial. Biceps strength is usually preserved relative to triceps in neurological conditions, but biceps has a larger cortical representation and might be expected to show preferential weakness in ALS.

**Methods:**

Using the South-East England Register for Amyotrophic Lateral Sclerosis, we performed a retrospective longitudinal cohort study and extracted the modified Medical Research Council (MRC) muscle strength score for biceps and triceps in patients with a diagnosis of upper limb-onset ALS in the 19-year period 1996–2015. A Wilcoxon signed-rank test was used to assess the relative strength of the muscles within the total sum of the upper limbs involved in the study.

**Results:**

There were 659 people with upper limb onset of weakness. In 215 there were insufficient data to perform the analysis, and a further 33 were excluded for other reasons, leaving 411 for analysis. Biceps was stronger than triceps in 87 limbs, and triceps was stronger than biceps in 258 limbs, with no difference seen in the remaining 477. Triceps strength scores (mean rank=186.1) were higher than ipsilateral biceps strength scores (mean rank=134.2), Z=−10.1, p<0.001 (two-tailed).

**Conclusion:**

Triceps strength is relatively preserved compared with biceps in ALS. This is consistent with a broadly corticofugal hypothesis of selective vulnerability, in which susceptibility might be associated with larger cortical representation.

## Introduction

Amyotrophic lateral sclerosis (ALS) is a neurodegenerative syndrome of the upper and lower motor neurons (and their wider cortical networks) characterised by relentlessly progressive skeletal muscle paralysis that ultimately leads to respiratory failure and death.^1^ The site of onset of clinical symptoms and their spread in ALS are non-random, but within a strikingly heterogeneous clinical syndrome that is currently unified by the postmortem identification of neuronal and glial cytoplasmic inclusions of Trans-Activating Response (TAR) DNA-Binding Protein-43 (TDP-43).

A well-recognised clinical phenomenon in ALS is the split hand sign, broadly characterised as preferential wasting and weakness of the thenar muscles, with relative sparing of the hypothenar muscles.^2^ More specifically, the sign is demonstrated by dominant wasting of the abductor pollicis brevis and the first dorsal interosseous on the lateral aspect of the hand, with sparing of the abductor digiti minimi on the medial aspect, resulting in the split phenomenon.^3 4^ This pattern of involvement is surprising, since isolated median nerve damage would not affect the first dorsal interosseous, ulnar nerve damage would not affect the thenar eminence yet should involve abductor digiti minimi, and root pathology would cause wasting of the thenar, hypothenar and interosseous muscles. Thus, the pattern cannot be explained by a lesion of the ulnar nerve, median nerve or root. One explanation for this phenomenon is that it reflects cerebral somatotopic hand representation, which is larger for the lateral hand for primates, in line with the evolution of fine motor control.^5 6^ The split hand would then reflect a greater vulnerability correlated to the size of the cortical map, supporting a broader concept of a corticofugal mode of neurodegeneration in ALS.^7 8^

A similar phenomenon exists at the level of the lower limbs in patients with ALS, characterised by preferential wasting of the ankle plantar flexor muscles compared with the dorsiflexors, resulting in a ‘split leg’ pattern.^9^ Although the ankle dorsiflexors receive a greater density of corticomotoneuronal projections than the plantar flexors, the nature of these projections with inhibitory cortical modulation being greater in the latter may help elucidate the pathogenesis in ALS in keeping with the hypothesis of altered inhibitory interneuronal function.^10^

A so-called pyramidal distribution of weakness is associated with lesions of the motor pathway anywhere from the cortex to the neuromuscular junction, whereby flexors dominate extensors in the upper limbs, and vice versa in the lower limbs. The elbow exhibits a degree of physiological flexion at rest, which is readily appreciated through the exaggeration that follows the maturation of a contralateral hemispheric stroke. However, the cortical representation of biceps is greater than that of triceps.^11^ For ALS, therefore, in keeping with the split hand and the split leg, one would expect biceps brachii to become preferentially involved reflecting its larger cortical representation. We therefore tested this hypothesis by examining the relative strength of biceps and triceps muscles in people with ALS.

## Methods

### Patient data

Data were gathered from the South-East England Register for Amyotrophic Lateral Sclerosis (SEALS). This population-based register was established in 1997 and captures every person with ALS within a defined geographical region including Kent and South East London.^12^ To ensure involvement of the upper limbs, we selected all patients with upper limb-onset ALS and used Medical Research Council (MRC) motor strength scores for elbow flexion with the arm fully supinated (biceps) and elbow extension (triceps) recorded at the first visit to the tertiary referral clinic. The MRC system grades muscle strength as 0 (no movement at all), 1 (a flicker of movement), 2 (movement of the limb, but not able to lift its own weight), 3 (limb able to lift its own weight but not against resistance), 4 (reduced power against resistance) and 5 (full power). Grade 4 is further subdivided into 4− (barely able to resist), 4 (weak) and 4+ (only a little weakness). We noted handedness and converted the modified MRC score given for each muscle to a scaled score of 0–7; MRC scores of 0, 1, 2, 3, 4−, 4, 4+ and 5 were mapped to the scores 0, 1, 2, 3, 4, 5, 6 and 7, respectively.

### Statistical analysis

As MRC scores are not normally distributed, the Wilcoxon signed-rank test was used to compare biceps power with triceps power in the same limb. Each limb was considered independently and not paired within the same individual. A sensitivity analysis was performed by excluding limbs in which both muscles had full power.

The binomial test of proportions was used to test whether differential strength patterns in one limb affected the probability of a similar observation in the other limb, and to test the degree of concordance between handedness and differential strength patterns.

The power function of the Wilcoxon signed-rank test is difficult to define.^13^ Using the exact variance approach, there is 80% power to detect a difference of 10% between biceps and triceps scores in a sample size of n=419, where n is the total number of upper limbs.

Data were entered into a spreadsheet, and calculations were performed using Excel and IBM Statistics SPSS V.24.

## Results

**Figure 1:**
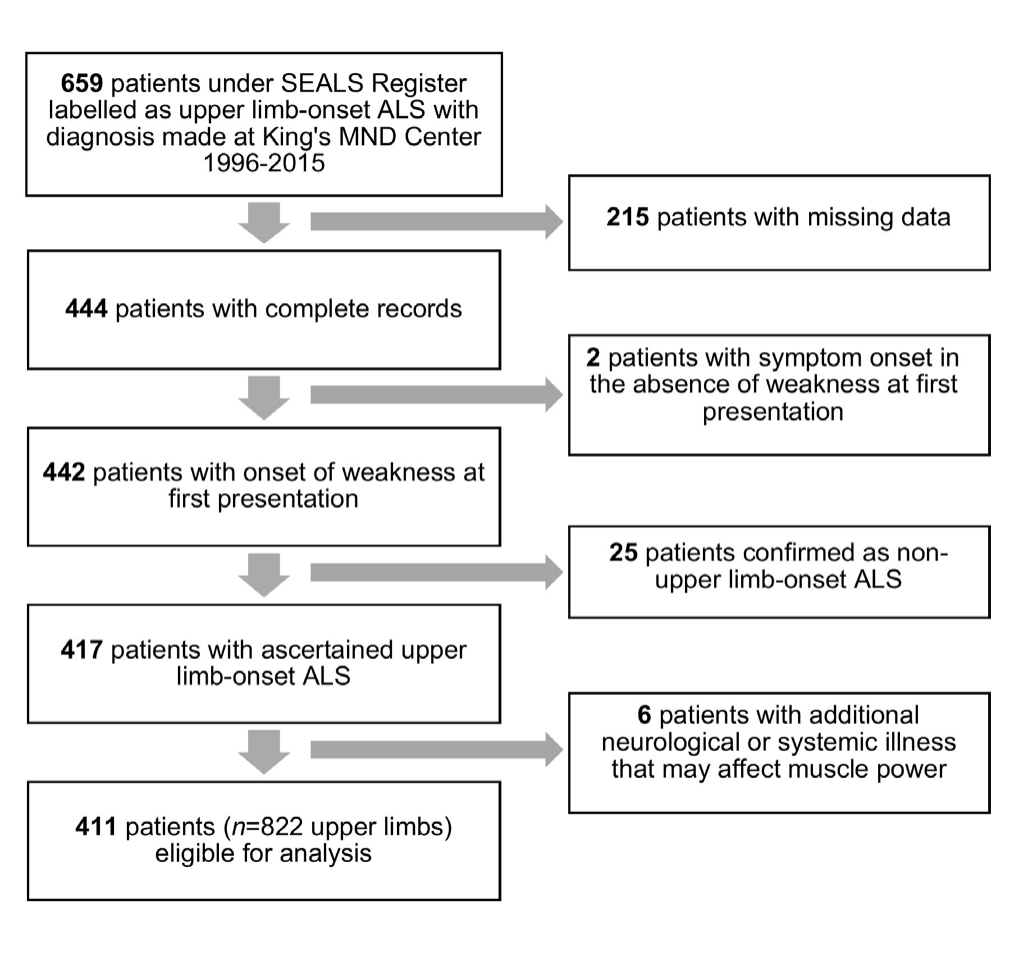
Flow chart illustrating the selection of eligible patients for the study. ALS, amyotrophic lateral sclerosis; MND, motor neuron disease; SEALS, South-East England Register for Amyotrophic Lateral Sclerosis.

In the SEALS register, there were 659 people recorded as having upper limb-onset ALS in the 19-year period from January 1996 to November 2015 (figure 1 shows a Consolidated Standards of Reporting Trials diagram). Detailed health records were missing for 215. Two people presenting with fasciculations or sensory symptoms in the absence of weakness were excluded. Review of the health records showed that 18 people in fact had lower limb onset and 7 had bulbar onset. Those with comorbidities that could influence MRC scores were removed from the subsequent analysis; these included spinal cord transection, polymicrogyria, peripheral neuropathy of undetermined cause, stroke, systemic lupus erythematosus and capsulitis. A total of 411 people and 822 upper limb score sets were therefore available for study.

The mean age of onset was 57 years (SD 13) and 74% were male. There was no difference in age with respect to gender. The initial upper limb weakness was right-sided in 41%, left-sided in 39%, bilateral in 19% and missing in 1%. There were 128 (31%) people previously or currently on riluzole. Eighty-five per cent were right-handed, 8% left-handed and 2% ambidextrous, with 7% missing handedness data.

**Figure 2:**
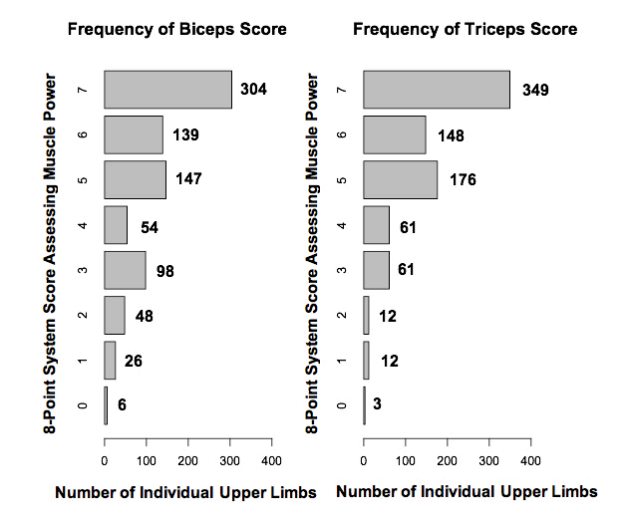
Bar charts indicating the frequency of scores for each limb on the 8-point grading system for both biceps and triceps.

Biceps was stronger than triceps in 87 limbs, triceps stronger than biceps in 258 limbs, and 477 limbs had equal scores. Overall, triceps was stronger than biceps (triceps mean rank 186.1, biceps mean rank 134.2; Z −10.1, p=8.5×10^−24^ [two-tailed]). The median triceps score was 6 (IQR 5–7) and the mean was 5.69 (SD 1.5, 95% CI 5.59 to 5.79), while the median biceps score was 6 (IQR 4–7) and the mean was 5.27 (SD 1.83, 95% CI 5.13 to 5.39). Frequencies for each score are shown in figure 2.

Limbs were analysed independently of the patient. This approach assumes that the pattern of weakness on one side is independent of the pattern of weakness on the other in the same person. We therefore tested this assumption. There were 88 people with concordant findings of stronger triceps in both limbs, and 82 with discordant findings in which only one limb showed the stronger triceps pattern. Assuming that it is equally likely that biceps or triceps will be stronger, a binomial test has p=0.701 (two-tailed), suggesting that the presence of the sign on one side does not influence its occurrence in the contralateral upper limb.

Of the 477 limbs with equal scores, 265 had full power. Repeating the analysis with these limbs excluded made no difference to the findings.

We explored the effect of handedness on the findings. Of the 258 limbs with triceps stronger than biceps, there was missing information on handedness for 15 limbs, leaving 243 for study. Of these, 78 had one side with triceps stronger and therefore available for analysis. There was no concordance between handedness and the side of stronger triceps: 45 concordant of 78 (58%), p=0.213 (two-tailed).

## Discussion

We have shown that, on average, triceps is stronger than biceps in upper limb-onset ALS, contrary to the classical so-called pyramidal pattern of weakness. If, as suggested in relation to the split hand and split leg phenomena, the explanation is that greater cortical representation of one set of muscles versus another makes the motor neurons more vulnerable to degeneration, then the study lends support to the broader concept of a cortically based process, and therefore a ‘split elbow’.

Our sample was typical of other similar samples, with 74% male as expected for spinal-onset ALS.^14^ Symptom onset in the limbs is usually asymmetrical, with the onset of weakness more frequently occurring in the dominant upper limb.^15 16^ While we reproduced that finding here, where the upper limbs were discordant for the split elbow, it was not more frequent on the dominant side.

The triceps muscles are innervated by the radial nerve, while the biceps muscles receive innervation from the musculocutaneous nerve; both nerves branch from the brachial plexus, which is collectively formed by the nerve roots C5–T1.^17^ In healthy individuals, the elbow flexors are stronger than the extensors, with average elbow flexion strength being 70.8 Nm in men and 39.3 Nm in women, and extension strength being 33.6 Nm in men and 19.3 Nm in women.^18^ A reduction in both flexion and extension by 30% is necessary to assess for a pyramidal distribution of weakness,^19^ and we would therefore expect that biceps would, on average, be stronger than triceps in ALS.

Attempts to distil the increasing complexity of the clinicopathological syndrome of ALS into either a peripheral ‘dying back’ or a central ‘dying forward’ process continue.^7 20^ Transcranial magnetic stimulation studies have shown hyperexcitability of the cortex as a perisymptomatic feature of ALS,^21 22^ and dying forward would mean that areas with higher cortical representation might be involved preferentially in neurodegeneration. Comparative observations of deep tendon reflexes between pairs of muscles might provide further support for a corticofugal pattern of degeneration. In this context, it is interesting that bulbar-onset ALS is characterised by preferential wasting of the tongue compared with other oropharyngeal muscles, as the tongue receives greater cortical innervation.^23^

Our study has limitations. Not all those identified as eligible for the study could be included because of missing health records, which will introduce bias into the study. The modified MRC score is vulnerable to differences in inter-rater and intrarater reliability, as well as intersubject variability.^24 25^ All our measurements were made by four specialists in ALS, trained by a single person, which would to some extent mitigate this issue, but a more objective measure of strength such as dynamometry would be ideal.^26–28^ This study is cross-sectional and therefore susceptible to the bias of prevalent, younger populations that attend clinic that is not present in population-based studies. It may be that the phenomenon observed is more likely to be seen in younger people, for example, and is not a general feature of ALS.

Replication of our findings in other cohorts is needed. The split hand phenomenon has been studied electrophysiologically to demonstrate its sensitivity and specificity as an early diagnostic tool, and similar studies would be important for the split elbow sign.^29^ Transcranial magnetic stimulation studies can serve as an objective assessment of upper motor neuron involvement in ALS and therefore cortical dysfunction, as has been shown with the split hand phenomenon,^30 31^ providing support for the hypothesis of a corticofugal mode of degeneration. Limiting the study by disease stage might also be useful because information is only available when there is some weakness but not complete weakness.^32 33^

The pattern of preferential weakness of triceps versus biceps we have found supports the broader concept of ALS as a neurodegenerative disease with a cortically defined pathogenesis.
